# A randomized phase II study of pelareorep and docetaxel or docetaxel alone in men with metastatic castration resistant prostate cancer: CCTG study IND 209

**DOI:** 10.18632/oncotarget.24263

**Published:** 2018-01-17

**Authors:** Bernhard Josef Eigl, Kim Chi, Dongsheng Tu, Sebastien J. Hotte, Eric Winquist, Christopher M. Booth, Christina Canil, Kylea Potvin, Richard Gregg, Scott North, Muhammad Zulfiqar, Susan Ellard, Joseph Dean Ruether, Lyly Le, A. Saranya Kakumanu, Mohammad Salim, Alison L. Allan, Harriet Feilotter, Ashley Theis, Lesley Seymour

**Affiliations:** ^1^ BC Cancer Agency, Vancouver, BC, Canada; ^2^ Canadian Cancer Trials Group, Kingston, ON, Canada; ^3^ Juravinski Cancer Centre, Hamilton, ON, Canada; ^4^ London Health Sciences Centre, London, ON, Canada; ^5^ Cancer Centre of Southeastern Ontario, Kingston, ON, Canada; ^6^ Ottawa Regional Cancer Center, Ottawa, ON, Canada; ^7^ Cross Cancer Centre, Edmonton, AB, Canada; ^8^ BC Cancer Agency, Kelowna, BC, Canada; ^9^ Tom Baker Cancer Centre, Calgary, AB, Canada; ^10^ BC Cancer Agency, Surrey, BC, Canada; ^11^ Cancer Care Manitoba, Winnipeg, MB, Canada; ^12^ Regina Cancer Centre, Regina, SK, Canada

**Keywords:** prostate cancer, oncolytic viruses, chemotherapy, clinical trial

## Abstract

**Background:**

Pelareorep is an oncolytic virus with activity in many cancers including prostate. It has *in vitro* synergism with microtubule-targeted agents. We undertook a clinical trial evaluating pelareorep in mCRPC patients receiving docetaxel.

**Patients and Methods:**

In this randomized, open-label phase II study, patients received docetaxel 75mg/m^2^ on day 1 of a 21-day cycle and prednisone 5mg twice daily, in combination with pelareorep (arm A) or alone (arm B). The primary endpoint was 12 weeks lack of disease progression rate (LPD).

**Results:**

Eighty-five pts were randomized. Median age was 69, ECOG performance status was 0/1/2 in 31%/66%/3% of patients. Bone/regional lymph node/liver metastases were present in 98%/24%/6%. The median prognostic score was slightly higher in Arm A (144 vs. 129 p= 0.005). Adverse events were as expected but more prevalent in arm A. The 12-week LPD rate was 61% and 52.4% in arms A/B (p=0.51). Median survival was 19.1 on Arm A and 21.1 months on Arm B (HR 1.83; 95% CI 0.96 to 3.52; p=0.06). No survival benefit of pelareorep was found.

**Conclusion:**

Pelareorep with docetaxel was tolerable with comparable LPD in both arms but response and survival were inferior and so this combination does not merit further study.

## KEY MESSAGE

Pelareorep is an oncolytic virus presently under clinical evaluation in a variety of cancers. A randomized phase II trial of pelareorep with docetaxel in metastic castration resistant prostate cancer was undertaken. Our results show that the combination of docetaxel and pelareorep is not worthy of further evaluation in this setting.

## INTRODUCTION

Prostate cancer is the most frequently diagnosed serious cancer, and the second leading cause of cancer death in men [1]. For metastatic castration resistant prostate cancer (mCRPC), docetaxel remains an effective treatment [2], but its benefit is modest. Studies evaluating more effective combinations are warranted.

Pelareorep (Reolysin; Oncolytics Biotech Inc, Calgary, Canada), is a non-attenuated Dearing strain of the double-stranded RNA reovirus serotype 3. Although this is a ubiquitous virus that is not known to cause any serious illness [3], it was found to preferentially infect and exhibit cytotoxic effects on human cancer cells, especially those with activated Ras pathway [4–6]. Recent data suggests that pelareorep infection may also potentiate anti-tumor immune responses [7–9].

Pelareorep was shown to have *in vitro* activity against many cancers including several prostate cancer cell lines [4] and early studies showed significant *in vivo* activity in several prostate cancer xenograft models [10]. Intralesional pelareorep injections administered to a cohort of patients with organ-confined prostate cancer demonstrated antitumor activity, increased CD8 T-cell infiltration and increased prostate cancer apoptosis [10]. Sei et al have demonstrated that while pelareorep shows synergism with several agents in chemotherapy sensitive NSCLC cell lines, consistent synergism with taxanes was seen in all cell lines tested [11]. It was observed that the addition of paclitaxel to pelareorep prolonged mitotic arrest and increased the rate of progeny virion production. The proposed underlying mechanism of this synergism is due to viral propensity to exploit microtubules as sites of viral replication [12, 13] and so microtubule stabilizing agents such as taxanes are a natural choice in the evaluation of combination therapies with pelareorep.

A recently published phase I trial evaluating the combination of docetaxel with pelareorep in 24 patients demonstrated excellent tolerability [14]. Of 15 RECIST evaluable patients there was 1 CR, 3 PR and 10 patients with stable disease, 1 evaluable patient with prostate cancer had a 30% decline in PSA from baseline [14].

The combination of pelareorep with taxanes in prostate cancer is therefore an obvious area of interest. The Canadian Cancer Trials Group (CCTG) conducted a randomized phase II clinical trial to evaluate the tolerability and clinical activity of pelareorep and docetaxel in patients with mCRPC.

## PATIENTS AND METHODS

### Study design

This was a randomized, open label multicenter phase II trial of pelareorep and docetaxel/prednisone or docetaxel/prednisone alone in patients with mCRPC. The primary endpoint was lack of disease progression at 12-weeks (LPD), as per the Prostate Cancer Clinical Trials Working Group guidelines [15]. Secondary endpoints included change in PSA, safety, tolerability, objective response and response duration, and overall survival (OS). Enumeration of circulating tumor cells (CTC) at baseline and after 6 and 12 weeks was also undertaken. This study adhered to the Declaration of Helsinki and Good Clinical Practice. Local ethics committee approval was obtained by all participating institutions and all patients provided written informed consent. This study is registered in www.clinicaltrials.gov as NCT01619813.

### Patient selection

Patients had metastatic adenocarcinoma of the prostate with clinically or radiologically documented disease, radiographic or PSA progression and a castrate level of testosterone, a serum PSA of ≥ 5 μg/L, and an Eastern Cooperative Oncology Group (ECOG) performance status of 2 or less. Prior chemotherapy was not permitted, but prior treatment with hormonal agents (including abiraterone acetate or enzalutamide) or radiotherapy was, provided a minimum of 4 weeks had elapsed before enrollment. Patients were not eligible if they had a history of previous invasive cancer (except adequately treated non-melanoma skin cancer, or tumours curatively treated with no evidence of disease for > 5 years), brain metastases, other serious medical illness, or if they were receiving immunosuppressive therapy or had known HIV or active hepatitis B or C infection.

### Treatment plan and evaluations

Patients were randomly assigned to receive either docetaxel at 75 mg/m^2^ IV on day 1 and prednisone 5mg orally twice daily with pelareorep at a dose of 3×10^10^ TCID_50_ intravenously daily on days 1 to 5 of a 3-week cycle (arm A), or docetaxel/prednisone alone (arm B). Docetaxel was delayed (up to 2 weeks) or reduced for toxicity, in accordance with the protocol. A single reduction to 1×10^10^ TCID_50_ was permitted for pelareoreop toxicity. Patients remained on treatment until protocol completion (10 cycles) or disease progression, intolerable toxicity, start of new cancer therapy, withdrawal of consent, or death. Baseline evaluation included physical exam, laboratory blood tests and radiological imaging with chest/abdominal/pelvic CT scan and bone scan. Laboratory tests for hematology, biochemistry and PSA were repeated on day 1 of every 3-week cycle. Radiological assessments were repeated at the end of every second cycle, and bone scan imaging was performed at 12 weeks, as clinically indicated and at end of study in patients with positive bone scans at baseline. CTC were collected at baseline [16], week 6 and week 12 if patients were still on study treatment. Adverse events were graded according to the NCI Common Terminology Criteria for Adverse Events (CTCAE) version 4.0.

### Outcome measures

All patients were considered assessable for toxicity from the time of their first treatment. Response and progression were evaluated by RECIST 1.1 criteria [17]. Bone scans were considered to show PD ONLY if > 2 new lesions were noted and confirmed by a second scan a minimum of 6 weeks later; the date of progression was the date of the first PD scan. PSA progression was evaluated following a minimum of 12 weeks on study and was defined as a rise in PSA of 25% (minimum 2 ng/mL) above baseline (or nadir) and confirmed by a second increasing value at least 3 weeks later. CTC counts were scored as favorable (<5 cells/7.5 mL blood) and unfavorable (≥5 cells/7.5 mL blood) at each measurement. Other endpoints included objective response rate in patients with measurable disease at baseline, overall survival and PSA change rate.

### Statistical considerations

The primary study endpoint was LPD at 12 weeks, determined by an algorithm based on PSA progression, bone scan progression and RECIST 1.1 progression as described above, as well as survival status. Secondary endpoints were objective response rate (ORR), defined according to the Modified Response Evaluation Criteria in Solid Tumours (RECIST 1.1), overall survival (OS), CTC counts, and potential prognostic or predictive molecular factors by assessment of archival tissue or blood samples.

With 40 patients treated by pelareorep plus docetaxel and prednisone, the study would have 92% power to test the null hypothesis that 12 week LPD rate was < 30% versus the alternative hypothesis that 12 week LPD rate was > 50% at 0.11 significance level. With a total sample size of 80, we would have 58% power to detect a difference in 12-week LPD rate from 30 to 50% with two-sided alpha 0.1 and 90% power to detect difference in 12 week lack of progression rate from 20 to 50% with two-sided alpha 0.1.

All comparisons between treatment arms were carried out at an alpha level of two-sided 10% unless otherwise specified. When appropriate, discrete variables were summarized with the number and proportion of subjects falling into each category, and compared using Fisher's exact test. Continuous and ordinal categorical variables were summarized using the mean, median, standard error, minimum and maximum values and when appropriate, compared using the Wilcoxon test. All confidence intervals were computed based on normal approximations.

All randomized patients were analyzed on an intention-to-treat basis for efficacy endpoints. Safety analyses included patients who had received at least one dose of protocol therapy.

Time-to-event endpoints (OS) were analyzed using Kaplan-Meier methods. Overall survival was calculated from the day of randomization to death. For alive patients at time of data cutoff, survival was censored as the last recorded date the patient was known alive. Primary comparisons of the treatment group were made using the stratified log-rank; sensitivity analyses adjusted for prespecified baseline factors (performance status [0 vs. 1-2], age [<65 vs. ≥ 65 years]). Hazard ratios (HR) with 80% confidence intervals (CI) were calculated from stratified Cox regression models with treatment group as the single factor. Discrete variables were compared using Fisher's exact test, and continuous ordinal categorical variables using the Wilcoxon test.

Circulating tumor cells were enumerated as previously described [16] at basline, week 6 and week 12 if patients were still on treatment (CELLSEARCH ®, Janssen Diagnostics, Raritan, NJ, USA). A favorable CTC status was defined as a CTC count < 5 CTC/7.5 mL.

Available archival tumour tissue also underwent mutational analysis as described in Supplemental Appendix 1.

## RESULTS

### Patient characteristics

**Figure 1:**
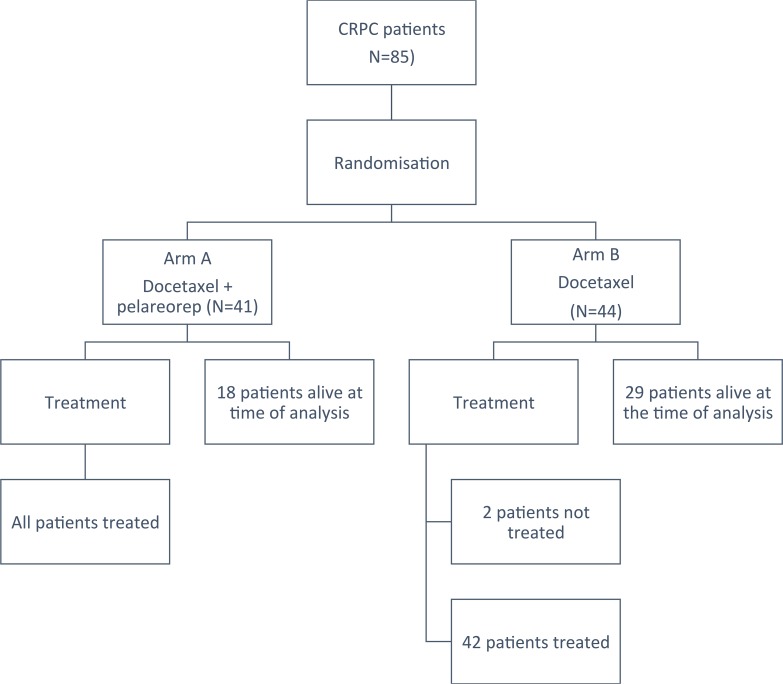
CONSORT diagram outlining subject disposition

Although protocol required accrual of 80 patients, eighty-five patients (41 on arm A and 44 on arm B; see Figure 1) were randomized from June 2012 to September 2015 at 11 centres because two patients on Arm B did not receive protocol treatment and 2 patients on Arm A and 1 on Arm B were found to be ineligible. All of these patients were followed for survival. Only patients who received protocol treatment were included in treatment exposure and safety analyses. Baseline characteristics are listed in Table 1. These were not significantly different between groups except for visceral disease status, LDH and alkaline phosphatase levels which were worse in Arm A. The prognostic model developed by Halabi *et al.* [18] was calculated, and the mean prognostic score between groups was respectively 145 and 129 on Arm A and Arm B (p=0.005).

**Table 1:** Baseline patient demographic and clinical characteristics

Characteristic		No. patients (%)		
		Arm A *N*=41	Arm B *N*=44	*P*-value
Age, years	Median	69.1	68.6	0.64
	Range	50.3 – 83.7	49.7 – 86.6	
ECOG Performance Status	0	8 (19.5)	18 (40.9)	0.07
	1	31 (75.6)	25 (56.8)	
	2	2 (4.9)	1 (2.3)	
Gleason Score	<8	13 (31.7)	12 (26.2)	0.92
	8-10	26 (63.4)	21 (68.9)	
	unknown	2 (4.9)	1 (2.3)	
Measurable Disease	No	26 (63.4)	21 (47.7)	0.19
	Yes	15 (36.6)	23 (52.3)	
Visceral disease	No	23 (56.1)	32 (72.7)	0.009
	Yes	18 (43.9)	12 (27.3)	
Months from relapse to randomization	Median	38.1	58.7	0.18
	Range	3.7 – 148.2	7.0 – 126.7	
Type of Progression	Radiologic	18 (43.9)	17 (38.6)	0.66
	PSA alone	23 (56.1)	27 (61.4)	
Prior Therapies	Androgen Ablation	41 (100)	44 (100)	0.90
	Abiraterone Acetate	15 (36.6)	13 (29.5)	
	Enzalutamide	9 (22.0)	9 (20.5)	
Number of Disease Sites	1	27 (65.9)	18 (40.9)	0.06
	2	8 (19.5)	18 (40.9)	
	3 or more	6 (14.6)	8 (18.2)	
PSA (ng/mL)	Mean (SD)	189.7 (266.8)	257.1 (473.1)	0.92
LDH	Mean (SD)	268.8 (127.2)	236.6 (97.7)	0.04
	≤ ULN	16 (42.1)	29 (67.4)	0.03
	>ULN	22 (53.6)	14 (32.6)	
Alkaline Phosphatase	Mean (SD)	389.4 (590.2)	214.3 (301.1)	0.01
	≤ ULN	14 (34.1)	24 (54.5)	0.08
	>ULN	27 (65.9)	20 (45.5)	
Hemoglobin	Mean (SD)	12.4 (1.6)	12.7 (1.5)	0.32
	≤ LLN	10 (24.4)	15 (34.1)	0.35
	>LLN	31 (75.6)	29 (65.9)	
Baseline CTC Result				
	Favorable (<5)	17 (41.5)	20 (45.5)	0.67
	Unfavorable (≥5)	22 (53.7)	20 (45.5)	
	Missing	2 (4.9)	4 (9.1)	
Prognostic score	Mean (SD)	144 (141)	129 (127)	0.005

### Treatments administered

More than half of patients received 7 cycles of treatment, with the median number of cycles being 7 for arm A (range 1 to 10) and 9 for arm B (range 1 to 13). The median cumulative docetaxel dose was lower in Arm A (457.4 mg/m^2^ vs 603 mg/m^2^). Thirty patients (73.2%) on Arm A had all administrations of pelareorep according to protocol, and 68.3% of patients received 90% or more of the planned dose. Patients in Arm A were more likely to discontinue docetaxel therapy prior to completion of all planned cycles (70.7 % vs 61.9%), predominantly because of disease progression. Docetaxel discontinuation due to toxicity was similar in both arms (11 vs 9 patients). More patients on Arm A required docetaxel dose modifications (56% vs 29%), with febrile neutropenia and fatigue being the major contributing factors.

### Safety

Adverse events with an incidence of >10% are listed in Table 2. These were generally grade 1-2 and in keeping with known toxicities for these agents. The incidence of acute grade 3 or worse events found in 5% or more in one or both treatment arms included (Table 3): febrile neutropenia, diarrhea, nausea, fatigue, and peripheral neuropathy. The incidence of non-neutropenic fever in arm A was 46% (44% attributed to reovirus) vs. 7% in arm B. Of febrile neutropenic episodes, 10% were related to reovirus while 27% (arm A) and 31% (arm B) were related to docetaxel. No grade 5 adverse events were observed.

**Table 2:** Adverse events occurring in >10% of patients in one of arms

Adverse event	Number of patients (%) with an event of any grade	
	Arm A (n=41)	Arm B (n=42)
Febrile neutropenia	11 (27)	13 (31)
Watering eyes	8 (20)	10 (24)
Abdominal pain	8 (20)	3 (7)
Constipation	17 (41)	19 (45)
Diarrhea	30 (73)	19 (45)
Dry mouth	5 (13)	3 (7)
Dyspepsia	8 (20)	8 (19)
Mucositis oral	22 (54)	21 (50)
Nausea	28 (68)	21 (50)
Vomiting	18 (44)	11 (26)
Chills	15 (37)	3 (7)
Edema limbs	18 (44)	19 (45)
Fatigue	40 (98)	40 (95)
Fever	19 (46)	5 (12)
Flu-like symptoms	12 (29)	1 (2)
Skin Infection	4 (10)	5 (12)
Upper respiratory infection	7 (17)	5 (12)
Bruising	6 (15)	6 (14)
Anorexia	27 (66)	21 (50)
Dehydration	7 (17)	2(5)
Arthralgia	15 (37)	9 (21)
Back pain	16 (39)	20 (48)
Bone pain	15 (37)	12 (29)
Generalized muscle weakness	11 (27)	6 (14)
Muscle weakness lower limb	8 (20)	10 (24)
Myalgia	11 (27)	11 (26)
Pain in extremity	18 (44)	12 (29)
Dizziness	12 (29)	6 (14)
Dysgeusia	21 (51)	24 (57)
Headache	16 (39)	7 (17)
Peripheral sensory neuropathy	18 (44)	28 (67)
Anxiety	3 (7)	5 (12)
Insomnia	17 (41)	14 (33)
Hematuria	5 (12)	3 (7)
Urinary frequency	23 (56)	17 (40)
Urinary incontinence	5 (12)	4 (10)
Erectile dysfunction	9 (22)	10 (24)
Cough	10 (24)	13 (31)
Dyspnea	19 (46)	17 (40)
Epistaxis	4 (10)	5 (12)
Productive cough	8 (20)	3 (7)
Sore throat	3 (7)	6 (14)
Alopecia	33 (80)	30 (71)
Dry skin	4 (10)	5 (12)
Hyperhidrosis	5 (12)	4 (10)
Nail discoloration	6 (15)	5 (12)
Rash maculo-papular	7 (17)	1 (2)
Hot flashes	19 (46)	19 (45)
Hypertension	5 (12)	7 (17)
Hypotension	8 (20)	3 (7)

**Table 3:** Adverse events grade ≥3 in >5% of patients

Adverse event	Number of patients (%) with grade ≥3 event	
	Arm A N=41	Arm B N=42
Febrile neutropenia	11 (27)	13 (31)
Diarrhea	4 (10)	0 (0)
Nausea	3 (7)	0 (0)
Fatigue	8 (20)	6 (14)
Sepsis	3 (7)	0 (0)
Peripheral sensory neuropathy	2 (5)	3 (7)
Syncope	4 (10)	3 (7)
Erectile dysfunction	7 (17)	6 (14)
Hypertension	2 (5)	4 (10)
Hypotension	3 (7)	3 (7)
Thromboembolic event	1 (2)	3(7)
Anemia	6 (15)	2 (5)
Elevated Alkaline Phosphatase	7 (17)	3 (7)

### Efficacy

The 12-week LPD rate was 61% for arm A and 52.4% for arm B and this was not statistically significant (p=0.51). Thirty-five patients were evaluable for objective response; response rates were higher in arm B (26.7% vs 40%; adjusted OR 0.53; 95% CI 0.12 to 2.38, p=0.41). CTC enumeration revealed no significant differences or changes between arms Supplementary Table 1.

**Figure 2:**
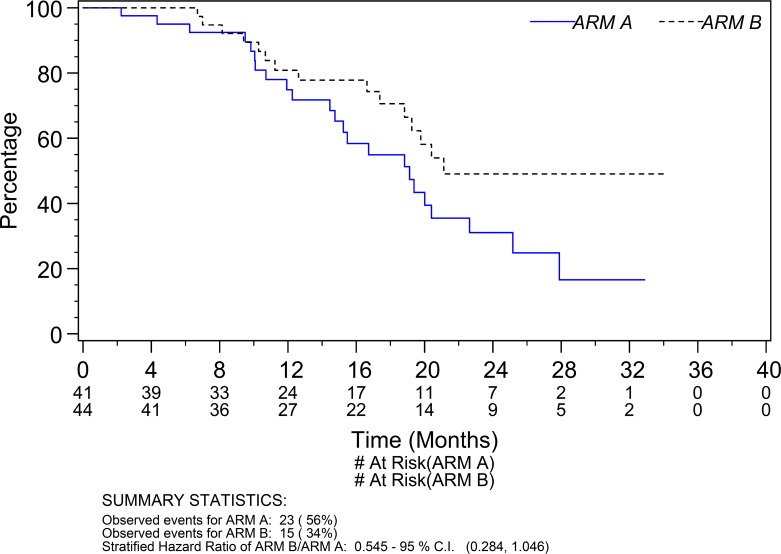
Overall survival

A total of 38 deaths (23 Arm A; 15 Arm B) were observed at the time of data cut-off. The median survival was respectively 19.1 months (95% CI 14.8 to 22.6 months) for patients on Arm A and 21.1 months (95% CI 18.8 to undefined) on Arm B (Figure 2). The hazard ratio for survival of Arm A to Arm B was 1.83 (95% CI 0.96 to 3.52; p=0.06). After adjusting for the potential prognostic factors prespecified in the analysis plan the adjusted hazard ratio of Arm A to Arm B was 1.86 (95% CI 0.97 to 3.58; p=0.06). In a sensitivity analysis adjusting for age and a prognostic score calculated based on baseline performance status, Gleason score, LDH level, alkaline phosphatase level, PSA level, hemoglobin level, and presence of visceral disease, the adjusted hazard ratio of Arm A to Arm B was 1.95 (95% CI 0.95 to 4.01; p=0.07). The median prognostic score was associated significantly with survival (adjusted hazard ratio of Arm A to Arm B was 7.55 with a 95% CI 1.09 to 52.2; p=0.04).

## DISCUSSION

The rationale for combining pelareorep with a taxane in CRPC was based upon the observations that: pelareorep has documented activity against prostate cancer cell lines [4], and in intratumoral injections in humans [10]; and that, in keeping with the proposed mechanism of viral replication occurring at microtubules [12, 13] consistent pelreorep synergism with microtubule stabilizing agents such as taxanes is observed *in vitro* [11].

Unfortunately, the results show that this combination has no evidence of activity worthy of further study. While OS was not a primary endpoint, note must be made of a trend toward worse survival in the combination arm. Several factors may have contributed to this. First, not unexpected in a small, albeit randomized, study there were some imbalances in baseline prognosic factors favoring arm B. It is unlikely that this minor imbalance would have overcome a promising signal of activity for arm A, and the strong trend to inferior survival remained after adjusted and sensitivity analyses. Second, this is the first study where pelareorep has been combined with daily steroids at potentially immunosuppressive doses. While there is some data that pelareorep may enhance immune-mediated anti-tumor effects [19], it has not been established whether this is the case for prostate cancer nor whether low-dose prednisone may have adversely affected any anti-tumor effect of pelareorep. Third, patients on arm A received fewer cycles and a lower dose intensity of docetaxel compared to arm B. The reason for this is not clear; discontinuation due to toxicity was similar in both arms (11 vs 9 patients), but more patients on Arm A required early docetaxel dose modifications due to toxicities. Certainly there are data supporting a decrease in outcomes for patients who received less than the standard dose intensity of chemotherapy [20]. While this might be ameliorated through dosing schedules avoiding virus exposure at neutropenic points in a cycle, the fact remains that the combination as tested was either not feasible to deliver, or not more efficacious than docetaxel. Finally, our study was a small RCT and was not powered to detect OS differences.

This study was one of four randomized phase II trials run by the CCTG that evaluated pelareorep in the metastatic setting (NCT01622543, NCT01708993, NCT01656538). In the two trials enrolling both genders, men appeared to fare worse than women, suggesting a possible impact of gender on outcomes [21], which may reflect on proposed gender based differences in immunity and response to immune-targeted therapies [22, 23]. Indeed there is an increasing body of evidence that oncolytic viruses are potent stimulators of immune activation, neo-antigen presentation, and PD-1/PD-L1 over-expression [24], Clinical trials evaluating pelareorep with checkpoint inhibitors are now planned or under way (NCT02620423).

In conclusion, while pelareorep given with docetaxel and prednisone is safe and tolerable in men with mCRPC this combination does not show activity worthy of further study in this setting.

## SUPPLEMENTARY MATERIALS TABLE


