# Supplementary material for: A randomized phase II study of pelareorep and docetaxel or docetaxel alone in men with metastatic castration resistant prostate cancer: CCTG study IND 209
Source: Oncotarget. 2018 Jan 17;9(8):8155–64. doi: 10.18632/oncotarget.24263 (PMC5814290; doi:10.18632/oncotarget.24263)
Supplement: Supplementary file 1 [file oncotarget-09-8155-s001.pdf]

A randomized phase II study of pelareorep and docetaxel or docetaxel alone in men with metastatic castration resistant prostate cancer: CCTG study IND 209

SUPPLEMENTARY MATERIALS

Supplementary Table 1: Circulating tumor cell favorable and conversion rate

	ARM A N=41	ARM B N=44	P Value
Circulating Tumor Cell (CTC)			
Baseline			.
N	39	40	
Median	5	4.5	
Range	0-139	0-253	0.65
Favorable (< 5 CTC/7.5 mL)	17 (41.5)	20 (45.5)	
Unfavorable (≥5 CTC/7.5 mL)	22 (53.7)	20 (45.5)	
Missing	2 (4.9)	4 (9.1)	
At 6 weeks			0.42
Favorable (< 5 CTC/7.5 mL)	23 (56.1)	28 (63.6)	
Unfavorable (≥5 CTC/7.5 mL)	11 (26.8)	8 (18.2)	
Missing	7 (17.1)	8 (18.2)	
At 12 weeks			0.55
Favorable ((< 5 CTC/7.5 mL)	22 (53.7)	25 (56.8)	
Unfavorable (≥5 CTC/7.5 mL)	9 (22.0)	6 (13.6)	
Missing	10 (24.4)	13 (29.5)	
Conversion from unfavorable to favorable			.
At 6 weeks	7 (17.1)	10 (22.7)	0.59
At 12 weeks	7 (17.1)	9 (20.5)	0.79
